# Challenging the principle of “No presumption of advantage” with a focus on testosterone and gender eligibility in sports

**DOI:** 10.3389/fspor.2025.1735032

**Published:** 2026-01-13

**Authors:** Philip J. Atherton, Ke Hu, Bernadette Jones-Freeman, Ada S. Cheung, Ricardo J. S. Costa, Nir Eynon, Jane T. Seto, Lukas Moesgaard, Morten Hostrup, Giscard Lima, Yannis Pitsiladis

**Affiliations:** 1 Faculty of Medicine & Health Sciences, Medical School, University of Nottingham, Royal Derby Hospital, Derby, United Kingdom; 2 Ritsmeikan Advanced Researcher Academy Fellow, Ritsumeikan, Kyoto, Japan; 3 Department of Sports and Health Sciences, Hong Kong Baptist University, Hong Kong, Hong Kong SAR, China; 4 Australian Regenerative Medicine Institute, Monash University, Melbourne, VIC, Australia; 5 Trans Health Research Group, Department of Medicine (Austin Health), The University of Melbourne, Melbourne, VIC, Australia; 6 Gender Clinic, Department of Endocrinology, Austin Health, Melbourne, VIC, Australia; 7 Department of Nutrition Dietetics & Food, Monash University, Melbourne, VIC, Australia; 8 Murdoch Children’s Research Institute, The Royal Children’s Hospital, Melbourne, VIC, Australia; 9 Department of Paediatrics, University of Melbourne, The Royal Children’s Hospital, Melbourne, VIC, Australia; 10 The August Krogh Section for Human and Molecular Physiology, Department of Nutrition, Exercise and Sports, University of Copenhagen, Denmark; 11 International Federation of Sports Medicine, Lausanne, Switzerland; 12 Department of Biology, Hong Kong Baptist University, Hong Kong, Hong Kong SAR, China; 13 Centre for Exercise Science and Medicine (CESAME), Hong Kong Baptist University, Kowloon Tong, Hong Kong SAR, China

**Keywords:** fairness, inclusion, performance, sexual development, testosterone

## Abstract

**Objectives:**

To evaluate the scientific validity of the International Olympic Committee's (IOC) 2021 framework principle of “No Presumption of Performance Advantage,” which suggests that circulating testosterone levels alone does not confer a competitive advantage.

**Design:**

A critical review of existing scientific literature concerning the physiological effects of testosterone and male puberty on athletic performance, complemented by a forward-looking proposal for integrating multi-layered biological and performance data.

**Method:**

Relevant peer-reviewed studies were examined, focusing on the role of endogenous testosterone, the long-term effects of male puberty, and performance outcomes among transgender women and athletes with differences in sexual development (DSD). Emphasis was placed on strength, power, and endurance metrics. In addition, we outline the emerging potential of combining wearable sensor monitoring with omics profiling to generate a comprehensive, dynamic evidence base.

**Results:**

Evidence consistently shows that male puberty is associated with lasting increases in muscle mass, strength, speed, and endurance due to elevated testosterone. Current evidence also suggests such physiological adaptations may endure even after testosterone suppression, potentially mediated by muscle memory mechanisms such as myonuclei permeance and/or epigenetic changes. If verified, transgender women who experienced male puberty and later reduced testosterone levels retain physical advantages, particularly in strength-based sports. Similarly, athletes with DSD exhibiting male-range testosterone levels also show performance advantages in select events.

**Conclusions:**

The IOC's principle of “No Presumption of Performance Advantage” contradicts a substantial body of scientific evidence. While inclusion and fairness are important goals, the framework's rejection of testosterone as a key determinant of athletic performance risks undermining competitive fairness and athlete safety in sex-segregated sports, particularly where strength and power are critical. Acknowledging testosterone as a key determinant of performance, our study proposes a novel multi-layered evaluation framework that integrates real-time sensor data with omics analyses. This innovative approach supports the development of adaptive, context-specific, and ethically grounded policies that promote fair, inclusive, and safe competition while offering a forward-looking roadmap for future policy and research.

## Introduction—the IOC framework on inclusion and fairness in sports

The IOC introduced a framework in November 2021 to promote fairness, inclusion, and non-discrimination on the basis of gender identity and sex variations in sports (1). Developed in response to growing global debates and high-profile cases involving transgender athletes and those with DSD, the framework represents a shift away from the 2015 recommendations of fixed circulating testosterone thresholds (2). Instead, it emphasises fairness and inclusion through individualised, evidence-based evaluations of multi-factorial indices, rather than rigid blanket one-size-fits-all criteria. As such, the new IOC Framework seeks to adopt a case-by-case approach, considering individual circumstances and guided by 10 overarching principles (Table 1) to ensure that all athletes, regardless of their biology, are given the opportunity to compete. These principles address key issues, such as, the prevention of harm, respect for privacy, and non-discrimination, while also grounding decisions in robust scientific evidence—vis-à-vis—data acquisition on physiological and psychological variables and informed decision (3).

**Table 1:** The 10 principles of the IOC framework (1).

Principle	Description
Inclusion	Everyone should be able to participate in sport without discrimination.
Prevention of Harm	Eligibility rules should not cause harm, physically or mentally.
Non-Discrimination	Rules must be fair and equitable for all athletes.
Fairness	Strives for competitive integrity while respecting athletes’ rights.
No Presumption of Advantage	There should not be an automatic assumption that certain physiological traits provide a performance advantage.
Evidence-Based Approach	Decisions should be grounded in robust and peer- reviewed scientific evidence.
Primacy of Health and Bodily Autonomy	Medically unnecessary procedures or treatments should not be performed solely for the purpose of meeting eligibility criteria
Stakeholder Involvement	Policies should be developed collaboratively, involving athletes, experts, and other stakeholders.
Respect for Privacy	Athletes’ personal and medical information must be handled confidentially.
Regular Review	Policies should be periodically reviewed to reflect the latest scientific and ethical understanding.

Among these principles, Principle 5, “No Presumption of Performance Advantage”, has attracted the most criticism (4, 5). This principle challenges traditional assumptions regarding physiological differences and their impact on sports and exercise performance (Table 2), such as the role of testosterone in altering physiological status that impacts strength, power, speed, and/or endurance capacity (6). Critics argue that the notion of simplistic assumptions about testosterone undermines competitive integrity, while proponents highlight its potential to create more inclusive policies that respect athletes’ identities and rights. Pertinent cases during the Paris 2024 Olympic Games, involving boxers Algeria's Imane Khelif and Taiwan's Lin Yu-Ting, have been viewed as violations of competitive integrity in light of Principle 5, “No Presumption of Performance Advantage.” As well as associated with elevated risk and therefore requiring robust safeguards in certain sport, such as boxing, where participants may realise fatal or life-changing injuries in the ring from blows to the head causing brain haemorrhage. It should be common sense that safety is of paramount consideration, already ahead of fairness and inclusivity in such sports. That is why boxing has weight divisions. This controversy has consequently damaged the IOC's reputation (7).

**Table 2:** The main components of the principle of “No presumption of performance advantage”.

Component	Description
Rejection of Simplistic Assumptions	The IOC asserts that testosterone levels alone should not be presumed to confer an automatic performance advantage
	Athletic ability is influenced by various factors, including training (i.e., load, recovery, and adaptation), skill, strategy, and mental resilience
Emphasis on Individual Assessment	Eligibility rules should be based on case-by-case evaluations rather than blanket policies
	Scientific evidence (i.e., data acquisition and interpretation) must demonstrate a significant, sport-specific performance advantage before imposing restrictions
Evolving Understanding of Performance	Research continues to challenge assumptions about gender-related advantages in sport, highlighting the complexity of physiological and non- physiological factors
Impact on Policy Development	This principle promotes a shift away from historical binary frameworks toward more nuanced policies that respect athletes’ identities and rights

**Table 3:** Representative quantitative findings from human studies of endogenous and exogenous testosterone and performance-related outcomes.

Study	Design	Duration	Population	Outcome
Endogenous				
West et al. (17)	RE	15 wks	Young men (*N* = 12)	MVC(*p* = 0.43), CSA(*p* = 0.27)
West et al. (16)	RE	12 wks	Young men (*N* = 56)	LBM (*r* = 0.14, *p* = 0.50); CSA (*r* = 0.09, *p* = 0.50; *r* = 0.08, *p* = 0.58); Leg Press (*r* = 0.06, *p* = 0.56)
Kvorning et al. (20)	3.6 mg goserelin vs. saline	8 wks	Young men (*N* = 22)	Isometric strength (goserelin vs. saline *p* = 0.05), LM (goserelin vs. saline *p* = 0.05)
Gharahdaghi et al. (21)	3.6 mg goserelin vs. saline	6 wks	Non-hypogonadal men (*N* = 16)	Strength (40 ± 2.3% vs. 49.8 ± 3.3%, *p* = 0.03); Muscle thickness (2.7 ± 0.4 to 2.69 ± 0.36 cm, *p* > 0.99 vs. 2.74 ± 0.32 to 2.91 ± 0.32 cm, *p* < 0.0,001); MPS (1.45 ± 0.11 to 1.50 ± 0.06%·day^−1^, *p* = 0.99 vs. 1.5 ± 0.12 to 2.0 ± 0.15%·day^−1^, *p* = 0.01); Muscle protein breakdown (93.16 ± 7.8 vs. 129.1 ± 13.8 g·day^−1^, *p* = 0.04)
Brook et al. (22)	RET	6 wks	Young men (*N* = 10) and old men (*N* = 10)	MVC (+21 ± 5%, *p* < 0.01), MPS (Y: 1.61 ± 0.1% day−1; O: 1.49 ± 0.1% day−1), and ribosomal biogenesis (RNA:DNA ratio and c-MYC induction: Y: +4 ± 2 fold change; O: +1.9 ± 1 fold change) increased only in young men
de Siqueira Guedes et al. (34)	240 mg GnRHa	3 wks	Healthy men (*N* = 30)	Creatinine and uric acid decrease; aromatic amino acids increase; cortisol decreases
Exogenous				
Hirschberg et al. (18)	10 mg testosterone cream vs. placebo	10 wks	Active healthy female (*N* = 48)	Running time to exhaustion increased 15.5 s more (*p* = 0.045); Greater total lean mass increased (*p* = 0.04)
Hirschberg et al. (19)	3, 6.25, 12.5, or 25 mg testosterone enanthate vs. placebo	24 wks	hysterectomized women (*N* = 24)	Increased paraspinal (1.26–6.88; *P* = 0.007), psoas (0.10–3.09; *P* = 0.038), and abdominal wall muscles (1.96–13.02; *P* = 0.011) CSA
Bhasin et al. (23)	25, 50, 125, 300, or 600 mg testosterone enanthate	20 wks	Eugonadal men (*n* = 54)	Thigh muscle (*r* = 0.66, *p* = 0.0,001) and quadriceps (r = 0.55, *p* = 0.0,001) muscle volumes correlated with log testosterone levels. Leg power correlated with log testosterone concentrations (*r* = 0.39, *p* = 0.0,105) and changes in fat-free mass (*r* = 0.30, *p* = 0.0,392) and muscle strength (*r* = 0.42, *p* = 0.0,020).
Bhasin et al. (24)	600 mg testosterone enanthate vs. placebo	10 wks	Healthy men (*N* = 43)	Triceps muscle size (424 ± 104 vs. −81 ± 109 mm^2^; *P* < 0.05); Quadriceps muscle size (607 ± 123 vs. −131 ± 111 mm^2^; *P* < 0.05); Bench-press (9 ± 4 vs. −1 ± 1 kg, *P* < 0.05); Squatting exercises (16 ± 4 vs. 3 ± 1 kg, *P* < 0.05)
Ferrando et al. (25)	200 mg testosterone enanthate	5 days	Healthy men (*N* = 7)	Protein synthesis increased 2-fold and reutilization of intracellular amino acids (*p* < 0.05)
Gharahdaghi et al. (29)	600 mg of testosterone enanthate vs. saline	6 wks	Non-hypogonadal healthy older men (*N* = 18)	mTOR, AKT, and RPS6: *p* < 0.05; Androgen receptor: 1.4-fold; Srd5a1: 1.6-fold; AKR1C3: 2.1-fold; HSD17*β*3: two-fold; IGF-1Ea (3.5-fold); IGF-1Ec(three-fold); Strength (*p* = 0.0,009), MVC (*p* = 0.002); FFM (*p* = 0.002)
Lima et al. (31)	Observational	NA	Non-trained men (*N* = 7) and resistance-trained who currently using AS (*N* = 19)	fibre CSA (8,160 ± 1,769 µm^2^ vs. 6,477 ± 1,271 µm^2^, *p* = 0.028)
de Siqueira Guedes et al. (34)	1,000 mg testosterone undecanoate	Single does	Healthy men (*N* = 30)	Creatinine and uric acid increase; aromatic amino acids decrease

As the IOC Framework signals a shift away from historical binary approaches (2), it also places a stronger emphasis on understanding the complex interplay of physiological and non-physiological factors influencing sport performance. Testosterone, a hormone with significant effects on musculoskeletal physiology, remains central to these debates (6, 8). As we explore this shift in policy, it is essential to address whether this is scientifically justifiable, particularly in the context of testosterone's role in muscle adaptation and sports performance. This review examines whether the IOC's 2021 ‘No Presumption of Advantage’ principle is scientifically supportable. To do so, we synthesize findings across endocrinology, applied physiology, and performance to evaluate how androgen exposure, sensitivity, and puberty-driven musculoskeletal adaptations shape sport performance and integrate recent developments in regulatory frameworks to highlight how evolving definitions of fairness and eligibility continue to reshape the ethical and therapeutic landscape of sport. Finally, we outline a forward-looking strategy that integrates sport-specific evaluation alongside emerging tools such as wearable sensor technologies and molecular profiling, to support an evidence-informed and context -dependent framework for advancing fairness, inclusion, and safety in elite sports.

## Methods

This manuscript was conducted as a structured critical narrative review, an approach suited to synthesizing heterogeneous evidence across exercise physiology, endocrinology, and sport performance, where systematic review and meta-analytic methods are not appropriate. The aim was to critically evaluate the scientific basis of the International Olympic Committee's (IOC) 2021 principle of “No Presumption of Performance Advantage” by integrating evidence on endogenous testosterone, male pubertal development, and athletic performance outcomes.

A targeted literature search was conducted using PubMed, Scopus, and Web of Science for studies published between 2000 and 2025. Searches focused on key conceptual domains including “testosterone”, “androgenic steroid”, “male puberty”, “resistance exercise”, “muscle mass”, “muscle strength”, “performance”, “transgender athletes”, and “differences in sexual development”. Boolean operators and combinations were applied to capture relevant studies across physiological, mechanistic, and performance-related domains. Primary emphasis was placed on peer-reviewed studies reporting measurable physiological or performance outcomes. Secondary sources, including animal studies, Court of Arbitration for Sport (CAS) case reports, policy statements, narrative and systematic reviews, were included to provide mechanistic, contextual, or governance-relevant information. Exclusion criteria included studies lacking relevance to performance or physiological outcomes, studies in populations outside the intended age or health range (e.g., pediatric non-pubertal populations), and articles not published in English.

Due to variability in populations, interventions, outcomes, and follow-up durations, quantitative meta-analysis was not performed. Instead, a qualitative synthesis was conducted, emphasizing directionality and consistency of effects, persistence of physiological adaptations following testosterone suppression, and mechanistic plausibility. Instead of using formal risk-of-bias tools, studies included were evaluated based on its design, sample size, how groups were compared, the reliability of the outcomes measured, and whether the findings were consistent with other studies.

## Testosterone and its role in muscle adaptation

Testosterone is a hormone synthesised primarily in the testes of males, and at greatly lower levels, the ovaries of females, with additional contributions from adrenal production occurring in the adrenal glands via androgen-precursors and peripheral conversion. The adrenal cortex also generates 11-oxygenated androgens (e.g., 11-ketotestosterone and 11-ketodihydrotestosterone), which can be peripherally converted into biologically active steroids, particularly in females. Together, these sources regulate diurnal circulatory testosterone levels. Prior to puberty, circulating testosterone levels are similar between boys and girls. Thereafter, serum testosterone markedly increases in males during puberty, with the testes producing 30-times more testosterone, such that circulating testosterone levels become ∼15–20 fold higher in males post-puberty, ranging 8–29 nmol/L in healthy males aged 18–40 years (as quantified by liquid chromatography-mass spectrometry). In healthy females, circulating testosterone levels peaks at 20–25 years and declines gradually with age but typically remains <2 nmol/L at all ages (6). Women with polycystic ovary syndrome (PCOS)^1^ or nonclassical adrenal hyperplasia exhibit mild hyperandrogenism, present higher levels of circulating testosterone [∼5 nmol/L (9)], albeit this represents the 99.99% confidence limit.

Testosterone is pivotal in regulating a variety of physiological processes, including muscle mass and function, most obviously during the process of male puberty. In males, testosterone secretion is regulated by the hypothalamic pituitary gonadal (HPG)^2^ axis, which operates through a classical positive-negative feedback loop that regulates its diurnal fluctuations. Instead, females produce substantially lower concentrations and the regulatory mechanisms governing production remains less understood, with no well-defined feedback loop akin to the HPG axis having been described. Nevertheless, despite the recognition of testosterone's critical role in male developmental biology, precise relationships between endogenous testosterone and human exercise-induced muscle adaptation remains a topic of contention, particularly in the context of other non-hormonal contributors, such as cellular mechano-transduction (i.e., those regulated at a tissue level). Several clinical experimental approaches have been employed to deconvolute the role of endogenous testosterone in exercise-induced muscle growth, which has a major biological role in resultant function and performance, with research focusing on, but not limited to, links between endogenous testosterone levels, including the impact and implication of post-exercise circulating concentrations (10–13).

How does exercise impact endogenous testosterone? It is notable that measured increases in circulatory testosterone concentration after exercise likely reflect decreased hepatic and whole-body metabolic clearance rates, rather than *de novo* production of testosterone induced by exercise, as has been shown in both males (14) and females (15). Therefore, acute serum testosterone induction by exercise does indeed seem an unlikely active mechanism in adaptation from the outset. For instance, acute post-resistance exercise (RE)^3^ elevations in serum testosterone concentrations were not associated with improvements in fat free mass, muscle fibre cross-sectional area (CSA)^4^, or strength after 12-weeks of resistance exercise training in recreationally active young men (16). It is technically noteworthy that acute concentrations of circulatory hormones can be influenced by many factors (e.g., timing of sampling etc), which will always be a limitation of such studies. In an elegant study using a different approach to address this conundrum, endogenous circulatory testosterone concentrations were physiologically manipulated by creating ‘high’ vs. ‘low’ hormonal environments via engagement of larger or smaller appendicular muscle groups, respectively (17). In doing so, it was observed that there were no differences in RE-induced muscle hypertrophy, strength gains, muscle-growth signalling pathways, or acute muscle protein synthesis (MPS)^5^ responses between repeated exposure to ‘higher’ vs. ‘lower’ hormone (including testosterone) conditions (17). These data suggest that acute post-exercise elevations in endogenous testosterone and resultant muscle exposure are unlikely determinants of muscle hypertrophy. Links between the low testosterone levels in female musculoskeletal physiology/exercise adaptation remain more so enigmatic. Circulating testosterone levels in females correlated with MPS and strength gains during RE, albeit to a lesser extent than in males (9). Similarly, data from longitudinal training studies have shown that higher baseline testosterone levels in pre-menopausal women were associated with enhanced muscle hypertrophy and strength improvements. It is highly likely that, in males or females, forging simple relationships at a single time-point with ensuing exercise-adaptations will lead to spurious conclusions. Moreover, and crucially, as frequently shown in males, but also in clinical trials in females (e.g., with low testosterone due to oophorectomy, and in pre-menopausal healthy women) exogenous testosterone provision increases bioavailability, muscle mass (18, 19), and aerobic capacity (18), exemplifying ergogenic effects. Considerations of endogenous vs. exogenous testosterone should thus not be conflated.

More controlled approaches to address the issue of endogenous testosterone's role in training adaptations and sports performance surround interventions aimed at clinically manipulating testosterone in the face of adaptation (e.g., inducing pharmacological hypogonadism or supplementing testosterone to assess the impacts on longer-term testosterone manipulation). In a landmark study addressing the impacts of testosterone depletion, Kvorning et al. conducted a randomised placebo-controlled trial in young healthy males, using the gonadotropin releasing hormone (GnRH^6^) analogue, Goserelin, to induce asynchrony in the HPG axis, thereby suppressing endogenous testosterone production (i.e., ∼23 to ∼1 nmol/L; mimicking clinical hypogonadism) during an 8-week RE intervention (20). Strikingly, the intervention resulted in ablation of improvements in maximal voluntary contraction, and attenuated muscle hypertrophy in comparison to the placebo group. These data highlighted the mechanistic role of testosterone sufficiency in strength-training adaptations and underlined how chronic sufficiency of testosterone does have a greater impact on muscle adaptation and performance outcomes than the passive transient acute increases (16, 17, 20).

In a more recent study of a mechanistic nature (21), non-hypogonadal men (18–30 years) were randomized in a double-blinded fashion to receive placebo (saline) or the injectable GnRH analogue, Goserelin (Zoladex, 3.6 mg), before 6-weeks of supervised whole-body coverage RE. This detailed mechanistic muscle biopsy trial showed that testosterone depletion, into the hypogonadal, but not fully depleted range, markedly blunted RE-induced muscle mass and individual and composite (appendicular) strength-gains. Likewise, testosterone depletion negatively impacted typical exercise-induced increases in MPS (as measured by deuterium oxide tracing across the RET period) that fundamentally underpin muscle hypertrophy. Synchronous to this was a blunted activation of molecular transducers of muscle hypertrophy on a cellular level, including typical androgen receptor upregulation, enhanced mechanistic target of rapamycin (mTOR)^7^-signalling and ribosomal biogenesis features, in addition to myogenesis biomarkers. The authors concluded endogenous testosterone sufficiency has a central role in the regulation of molecular transducers of RE-induced muscle adaptation in humans that cannot be overcome by, for example, muscle-level mechano-transduction. In sum, these core mechanistic human trials illustrate that non-hypogonadal levels of endogenous testosterone availability are a critical requirement for muscle hypertrophy, irrespective of acute exercise-associated increases in testosterone from which spurious associations may be derived.

Natural age-related declines in testosterone also present a model of the impacts of testosterone depletion in relation to exercise adaptation and taking into account confounding by other age-related changes. It is intuitive to the readership, but also factual that ageing is associated with dampened adaptive muscle hypertrophic potential, with research showing that exercise-induced muscle growth is attenuated in older people subjected to allied training programmes to younger group (22). This so-called “anabolic resistance” is now an accepted term for these deficits. In terms of exogenous testosterone (NB. synthetic isotopic distinctions are biologically irrelevant) it is irrefutable that DHT/testosterone analogues elicit anabolic effects, in both the absence (18, 23) and presence of an exercise stimulus (24). On a regulatory level, testosterone supplementation has dose-dependent effects on skeletal muscle mass, strength gains, and regulates key molecular pathways governing muscle hypertrophy that includes MPS (25, 26) satellite cell activation and proliferation (27, 28) in cohorts of younger and older individuals. To further elucidate the role of exogenous testosterone in exercise-induced muscle adaptation (on the back-drop of age-related anabolic resistance in ageing), Gharahdaghi et al. investigated the effects of bi-weekly testosterone injections (Sustanon 250 mg) in non-hypogonadal healthy older men (65–75 years) during a 6-week whole-body resistance exercise training intervention (29). It was reported that testosterone administration enhanced MPS muscle mass gains, and composite strength. Further evidence of enhanced MPS was provided by the recapitulation in activation of several molecular transducers (vs. placebo) of muscle hypertrophy in biopsy tissue, including androgen receptor upregulation, mTOR-signalling, ribosomal biogenesis, and myogenesis. These findings underscore that exogenous testosterone provision rescues typical upregulation of molecular transducers involved in resistance exercise training-induced muscle hypertrophy in older humans, and thus directly, implicates age-related declines in testosterone in the phenomenon of anabolic resistance. This further underlines the importance of testosterone adequacy in adaptation, *per se*.

## The long-term effects of testosterone exposure

In addition to the widely acknowledged benefits of testosterone for sports performance and the high prevalence of lifetime androgenic steroid (AS)^8^ use (30), a seminal study in 2013 from Gunderson's laboratory proposed that transient exposure to high circulating testosterone levels had long-term effects on hypertrophic responsiveness of skeletal muscle (31). It was shown that treatment with testosterone in mice for 14 days resulted in an increase in muscle size and fibre myonuclei number. However, during a 1-week washout period achieved by removal of the delivering implant, blood testosterone concentrations returned to baseline, and after 3 weeks the muscle CSAs were similar to baseline, while myonuclear numbers were retained for at least 3 months. Subsequent exposure to muscle overload for 6 days to induce muscle growth revealed that mice treated with testosterone displayed 31% greater hypertrophy than controls, indicating the presence of a cellular “memory”, or legacy preservation of pre-exposure in slow turning-over muscle myonuclei pools, with transient testosterone exposure.

Notably, not only do aforementioned animal studies provide valuable insights, but recent studies imply translational relevance. A study by Egner et al. demonstrated that the muscle memory phenomenon lasts 10% of the lifespan in mice (31); this is such that in humans, utilising 14C, this indicates an average age of the myonuclei of 15 years, suggesting the possibility of long-lasting implications for muscle memory (32). In line with this, when investigating data from previous AS users, an observational study reported similar myonuclei numbers between sedentary males, clean powerlifters, current and previous AS users. Yet, there were longitudinal decreases in muscle CSA with stable myonuclei number per fibre, aligning with the myonuclei maintenance premise in mice (31). Another cross-sectional study revealed a higher myonuclei density 4 years after AS cessation, further indicating this as a putative cellular mechanism, albeit in a small group of recreational strength trainees (33). While these findings need to be validated in human randomized-controlled trials, recent evidence points toward a myonuclei advantage and it follows an enhancement of metabolic regulation after a period of testosterone supplementation (34). If verified, this could have major implications for the development of sports policy making, not only with regards to individuals undertaking feminizing hormone replacement therapy or prior exposure to AS irrespective of biological underpinnings, but also for ensuing penalties following a positive test for androgen doping. However, myonuclei permanence is not fully established as merging evidence across species suggests that myonuclei are lost after a prolonged period of atrophy (35). The lack of difference in myonuclei number between sex in moderate trained young adults logically extends the uncertainty about myonuclei permanence (36). Whether muscle memory is a feature of prior muscle hypertrophy and/or testosterone *per se*, requires robust elucidation.

Even in the absence of definitive evidence, its potential implications of testosterone-associated muscle memory warrant careful consideration and should not be overlooked (Table 3). As such, scenarios where individuals are currently or have previously been exposed to higher levels of testosterone could present challenges where muscle mass substantially influences performance. Consequently, even after feminizing hormone replacement therapy lowers circulating testosterone levels, the hypothesized persistence of testosterone-associated adaptations has prompted discussion of potential implications for transgender females who underwent male puberty prior to transitioning, particularly in strength- and power-based sports, though these are typically regulated by weight categories that control for body mass–related advantages. These factors complicate discussions around fair competition in sports and underscore a need for tailored approaches that consider the long-term effects of early exposure.

## Testosterone levels as sole eligibility criteria in elite female competition

The Indian sprinter Dutee Chand was disqualified from taking part in the 2014 Commonwealth Games and Asian Games due to elevated testosterone levels (37). The Court of Arbitration for Sport (CAS)^9^ delivered an interim award partially upholding Chand's challenge and suspending the Hyperandrogenism regulations established by World Athletics [WA, formerly known as the International Association of Athletics Federations (IAAF)]^10^, raising concerns about the scientific validity of the Regulations. The panel of CAS countered that endogenous testosterone is not an appropriate criterion to explain the difference between male and female sport and exercise performance by noticing an overlap in testosterone concentration between sexes (37, 38). However, a subsequent study highlights that circulating testosterone in healthy males is ∼15-fold greater than typical females and contributes significantly to biological sex differences (39). The panel also noted that endogenous increases in testosterone do not lead to the same enhancement of skeletal muscle growth as exogenous increases (38, 40). Nonetheless, there is evidence indicating that increased muscle mass and strength in women with PCOS is associated with a mild increase in circulating testosterone (41). Likewise, the substantial increases in fat free mass during puberty in male adolescents is associated with serum testosterone levels (42), strongly suggesting a role of endogenous testosterone in muscle mass regulation. The panel even referred to published academic articles that disputed the connection between testosterone with sports performance (37, 43), despite the consensus of physiological function and physical performance significance of testosterone (44, 45). Given the absence of scientific evidence concerning the degree of performance advantage that hyperandrogenic females may enjoy (37). CAS concluded that every athlete should not be prevented from competing in any category as a consequence of the natural and unaltered state of their body.

To verify the quantitative relationship between enhanced testosterone levels and improved sports performance in hyperandrogenic athletes, the WA found that high free-testosterone levels provide a significant competitive advantage for female athletes in several track and field events that based on 2,127 observations during the 2011 and 2013 IAAF World Championships (46). However, the authors published a correction due to anomalies and errors including duplicated athletes, duplicated and phantom times identified during an independent scrutiny. In the corrected paper, 230 observations were excluded, resulting in a significant reduction of performance differences between high and low testosterone tertiles across 8 of the 11 events analysed (47). Of these 8 events, 3 showed a reversal in performance difference, changing from positive to negative where athletes with high testosterone levels performed slower. This dramatic change of results has once again put the new regulations in the spotlight (48), and the controversy over the eligibility of “female athletes with hyperandrogenism” continues. The term “female athletes with hyperandrogenism” has been used in sports regulations to describe individuals competing in the female category with elevated androgen levels, including both 46,XX females (e.g., with PCOS or congenital adrenal hyperplasia) and 46,XY individuals with DSDs. However, for scientific clarity, these groups differ biologically and should be distinguished in discussions of eligibility and performance.

Understanding the extent of performance declines when testosterone levels are pharmacologically attenuated becomes particularly significant surrounding the discussion about the eligibility of the female category. The reduction of circulating testosterone levels in transgender female athletes undergoing feminizing hormone replacement therapy represents a vulnerable view. A retrospective study evaluating the performances of 8 non-elite transgender female runners, showed statistically similar self-reported running times before and after their transitions (49). However, the findings of this study have not been replicated given the small sample size and lack of circulating testosterone concentration data. That physical fitness data from the U.S. Air Force demonstrated transgender women's physical performance declined by as much as 31% as testosterone levels decreased after 2 years of feminizing hormone therapy, which confirms the association between performance loss and diminished circulating testosterone levels (50). In a recent cross-sectional trial, researchers indirectly revealed the positive correlation between serum testosterone and physical performance by comparing transgender female athletes, whose hormone levels had decreased to those of cisgender females, with cisgender male athletes in terms of handgrip strength and lower body anaerobic power (51). A follow-up analysis showed declines in handgrip strength (7%–13%) and countermovement jump (23%–29%) after a year of suppressing the circulating testosterone concentration, reinforcing this correlation in a longitudinal study design (52).

The inclusion of transgender individuals in sports, especially in the context of feminizing hormone replacement therapy, also presents a complex challenge, particularly when considering the effects of feminizing hormone replacement therapy on muscle health and performance (53–57). The existing body of research is limited by small sample sizes, short follow-up periods, and inconsistent methodologies (58). Furthermore, transgender individuals often face exclusion from physical activities due to misconceptions about their performance capabilities and a lack of evidence-based policies (59, 60). A long-term cohort study by Jones et al. (61) is in place and aims to fill this gap by examining how hormone therapy influences muscle health and exercise-induced adaptations, by gaining a better understanding of how testosterone affects skeletal muscle function in transgender individuals through molecular analysis. Such research could contribute to fairer, evidence-driven policies in both clinical and sporting settings. This is particularly important given the growing number of transgender individuals seeking hormone therapy, and the urgent need for policies that reflect the physiological realities of their experiences (62). However, the recent implementation of SRY-based sex test by World Athletics and World Boxing introduces another blanket policy, which was deemed unscientific and unethical due to their oversimplification of sex determination and was abandoned before the Sydney 2000 Olympics (63, 64). The reintroduction of a historically discredited approach risks repeating past mistakes by imposing a simplistic, one-size-fits-all criterion, further highlighting the need for a nuanced, evidence-based eligibility framework that avoid such scientifically flawed approaches.

## Balancing fairness, inclusion, and safety

Testosterone plays a pivotal role in development, sports and contributes significantly to exercise performance, augmenting skeletal muscle mass, strength, power, and endurance-factors that are important determinants of athletic success across various sports. That said, sport performance is indelibly influenced by a complex interplay of individual variability (genes), sport-specific demands (bioinformatics), and non-hormonal factors (e.g., cellular communication, skill, training load and adaptations, and conditioning). The foundation of categorization in sport roots in relatively stable performance inequalities, while striving to achieve a balance that maximizes inclusion of diverse athletes and ensures the highest level of fairness. Consequently, policies governing athlete eligibility must move beyond rigid hormone thresholds *per se*. Instead, they should adopt a more sophisticated, objective, and evidence-based approach that prioritises fairness, inclusion, and safety, while considering how sex hormones, such as testosterone can influence performance characteristics.

**Figure 1:**
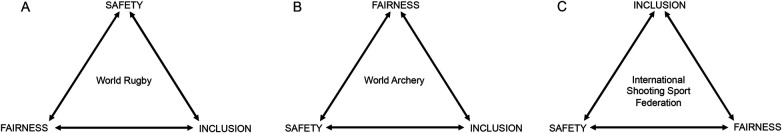
The decision-making triangle proposed by Hamilton et al. (65). **(A)** shows World Rugby with priorities: Safety at the top, Fairness and Inclusion at the base. **(B)** shows World Archery with Fairness at the top, Safety and Inclusion at the base. **(C)** shows International Shooting Sport Federation with Inclusion at the top, Safety and Fairness at the base. Arrows indicate the direction of priorities.

In line with this, Hamilton et al. (65) proposed a decision-making triangle that emphasised the need to balance safety, inclusion, and fairness when determining the eligibility of transgender athletes (Figure 1). The same idea could also be applied to athletes with DSD. Safety is a primary concern, as the physical well-being of athletes must be protected, particularly in sports with high physical impact demands, such as contact and combat sports. In rugby union, for example, differences in skeletal muscle mass, strength, power, and endurance capacity can pose significant risks, making it essential to consider the effect of hormone-driven changes during puberty to promote fairness among athletes, while also preventing and/or mitigating risk of potential injury. This concept extends to other combat sports, such as boxing, mixed martial arts, judo and/or wrestling, where the intensity and force of physical contact and the impact on athletes’ bodies necessitate stringent safety strategies. Alongside testosterone regulation, evidence-based frameworks incorporating biomechanical pre-evaluation, real-time physiological monitoring during competition and systematic medical screening following events could provide a dynamic approach to safeguarding both fairness and athlete health. In endurance sports, such as middle- and long-distance running, road cycling, and triathlon, with characteristics that include cardiorespiratory fitness, efficiency, and resilience play a more dominant role in performance than sheer strength, power, and impact tolerance. Here, fairness may need to take precedence over safety or inclusion, requiring some level of control to attempt levelling the playing field between diverse athletes. For precision sports like shooting and archery where skill, accuracy, and mental focus outweigh physical strength and power, fairness and safety concerns are less pronounced. In these sports, inclusion can be emphasised without the need for stringent hormonal regulation. The focus here would be on creating an environment where athletes, regardless of their gender identity or sex assigned at birth, can participate without undue restrictions, ensuring that their skill and dedication are the primary determinants of success.

Hamilton et al.'s (65) decision making triangle advocates a tailored approach to each sport, recognising each has its own unique demands and competitive structures. Sports reliant on strength, power and aggression such as weightlifting, boxing, rugby, require careful regulation of testosterone to ensure safety and fairness. By adopting policies that prioritise safety, inclusion, and fairness, and by implementing necessary medical interventions where gender classification in sport is required, sports organisations can create a more equitable and welcoming environment. This approach aims to avoid the exclusion of transgender or athletes with DSD, ensuring that their participation does not come at the expense of fair competition.

While medical interventions, such as testosterone suppression therapy, growth hormone therapy, feminizing hormone therapy, Erythropoiesis-stimulating agents and ADHD medication (e.g., stimulants like Adderall and Ritalin), may be necessary in some contexts to maintain fairness, they should be viewed as a therapeutic use exemption for fostering inclusion and equity, not as punitive or mandatory measures. Where medical care is indicated, only voluntary, evidence-based interventions consistent with ethical medical practice should be considered. For transgender athletes, gender-affirming hormone therapy (GAHT) often represents a medically indicated and personally chosen treatment; in contrast, medical interventions may not be desired or clinically warranted for athletes with DSD. In such cases, fairness should not be pursued through coercive medical requirements, but rather through individualized, sport-specific evaluation frameworks that consider the actual physiological relevance of each condition. Striking a balance between inclusion, competitive integrity, and safety requires an evidence-based strategy. Policymakers and sports organisations should therefore adopt inclusive, evidence-based eligibility models that recognize both the diversity of biological development and the need for equitable competition. Integrating longitudinal data on GAHT-induced muscle adaptations, alongside detailed analyses of DSD phenotypes and performance, can help determine where physiological advantages may meaningfully affect results across various sports. Such an approach respects athletes’ rights and autonomy while advancing fairness, safety, and integrity in sport.

## Future potential in harnessing technological advances for evidence-based policy making

While this narrative review synthesizes current evidence on testosterone-mediated adaptations and athletic performance, it is limited by the absence of quantitative synthesis and formal formal risk-of-bias scoring, meaning that findings are presented qualitatively and relative study weight is not formally assessed. Mechanistic insights provide valuable context but have limited immediate applicability for decision-making. To address these gaps and guide future research, we propose a conceptual roadmap integrating longitudinal monitoring, wearable sensor data, and multi-omics profiling to better understand persistent adaptations and inform evidence-based policy in sport.

Emerging in-competition monitoring technologies offer unprecedented opportunities to manage athlete safety and performance in real time. Wearable and embedded sensor systems, such as smart mouthguards that detect head impacts in contact sports like boxing and rugby, offer continuous, data-driven insights into physiological and biomechanical strain (66). When implemented ethically and with athlete consent, these tools provide actionable insights for concussion management, fatigue monitoring, injury prevention, and adaptive workload regulation, while supporting fairer and safer competition environments. Complementing real-time monitoring, advances in omics technologies provide an unparalleled opportunity to expand understanding of the long-term effects of testosterone on human physiology, performance and adaptation, and how human genetic variations influence the response to testosterone (67–69). These approaches hold the potential to address critical knowledge gaps, particularly regarding the molecular mechanisms underlying testosterone's influence on physiological adaptation, lingering effects and perhaps longer-term muscle memory and the development of evidence-based policies for sports and hormone use regulation.

Epigenomics examines both heritable and developed DNA modifications that influence gene expression without altering the underlying DNA sequence. Epigenetic modifications, such as DNA methylation and histone acetylation, can persist after the cessation of AS use, serving as molecular imprints of prior exposure (70–73). These effects may be mediated by changes in promoter methylation and chromatin accessibility of key muscle-regulatory genes, which in turn influence transcriptional activity during subsequent training. Complementing these insights, transcriptomics and proteomics enable the comprehensive analysis of gene expression and protein production, offering a window into the cellular processes influenced by testosterone. Testosterone is known to regulate the transcription of genes involved in muscle hypertrophy, repair, and metabolic function, such as those encoding myogenic regulatory factors and structural proteins like myosin and actin (74–76). Proteomic analyses further illuminate post-translational modifications that drive functional adaptations, including the mTOR signalling cascade, enhancing protein synthesis and cellular growth (77–79). Metabolomics provides an additional layer of biochemical consequences of testosterone fluctuations, revealing how testosterone profoundly shapes energy production, lipid oxidation, and amino acid metabolism, mitochondrial efficiency, and how these processes vary between individuals (80–84). Metabolomic profiling not only informs the biochemical mechanisms underpinning performance but may also highlight recovery capacity and responsiveness to training, linking physiological variability to competitive outcomes.

Despite the promise of these approaches, their application requires robust validation (and replication) through large-scale, longitudinal studies. Short-term, in-competition monitoring technologies, such as wearable sensors and smart mouthguards, can capture real-time data on workload, fatigue, and biomechanical stress, providing immediate, actionable insights for athlete safety and performance regulation. Complementing these short-term measures, multi-omics datasets, including epigenomic, transcriptomic, proteomic, and metabolomic analyses—offer a long-term perspective on how testosterone exposure shapes physiology, adaptation, and recovery. Integrating sensor-derived performance metrics with molecular and cellular data will be critical for uncovering causal links between hormonal exposure and functional outcomes, while accounting for genetic predisposition, environmental factors, and lifestyle variables, therefore, revolutionise our understanding of testosterone's role in physiology. The insights gained will not only inform evidence-based regulations in sports but also guide personalised interventions to optimise health, performance, and recovery while safeguarding long-term well-being.

## Conclusions

The IOC's 2021 Framework on Inclusion and Fairness in Sport marks a critical shift towards prioritising inclusivity and fairness through individualised assessments, moving beyond rigid testosterone thresholds. The complexities of testosterone levels, muscle biology, and sport performance require an evidence-based approach to athlete eligibility, particularly for transgender and athletes with DSD. Emerging evidence on the long-term effects of testosterone further highlights the importance of careful consideration in policy development.

Recognizing the persistent physiological effects of male puberty and testosterone, we outline a multi-layered conceptual framework that may help generate more sport-specific and empirically grounded evidence. In-competition monitoring and longitudinal molecular profiling represent promising tools for enhancing the resolution of performance, workload, and physiological data; however, their application remains largely theoretical. Further methodological development, validation, and interdisciplinary collaboration will be required before such approaches can meaningfully contribute to eligibility frameworks.

Navigating these challenges necessity a multidisciplinary, evidence-based framework that balances inclusivity, competitive integrity, and athlete safety, guided by ethical principles and continuous scientific evaluation. As the discourse continues to evolve, it is vital that regulations uphold fair competition while protecting every athlete's right for sports can remain a platform for excellence, inclusion, and equitable competition.

## Footnotes

^1^PCOS, polycystic ovary syndrome

^2^HPG, hypothalamic pituitary gonadal

^3^RE, resistance exercise

^4^CSA, cross-sectional area

^5^MPS, muscle protein synthesis

^6^GnRH, gonadotropin releasing hormone

^7^mTOR, mechanistic target of rapamycin

^8^AS, androgenic steroid

^9^CAS, court of arbitration for sport

^10^WA, world athletics; IAAF, international association of athletics federations
